# Report of Committee on President's Address

**Published:** 1893-02

**Authors:** 


### INTERNATIONAL HAHNEMANNIAN ASSOCIATION MEETING OF 1892.

---

*Third Day—Afternoon Session.*

Thursday, June 23d, 1892, 4 P. M.

### REPORT OF COMMITTEE ON PRESIDENT'S ADDRESS.

Your Committee congratulates the Association on the tenor and happy expressions contained in the President's address. We take his statements as to the number of homœopathists as his individually, and after some investigation, we are glad to report that in our opinion he is in error, especially as to the number located in the West. That we have difficulty in advising our patients as to whom they shall apply when traveling we all admit, but we are assured that the number of strictly homœopathic prescribers is sufficient did we but know them, and recommend that all Hahnemannian societies and *Organon* clubs be invited to send us their rolls of members as well as their Constitutions, and that after revision they be printed in our transactions for the information of members, and that our Corresponding Secretary also furnish us with the names of foreign physicians to whom we can safely intrust our patients. We congratulate the Association on the comparatively satisfactory growth of the organization, as shown by the President, and see in it a reason for exercising the same care as to the admission of members as has been practiced by the Board of Censors. We regret to recognize the truth of the charges of the President against the teachings of the Colleges, and recommend that each Alumnus in this Association use unceasing effort to secure characteristically homœopathic teaching in Materia Medica and Therapeutics in their respective colleges. We also congratulate the President upon his pleasant and fruitful connection with the institution mentioned in his address, and urge upon members the importance of accepting such positions as are offered to them in order that their influence may advance the cause of pure Homœopathy.

We all agree with the President and congratulate the Association that the spirit of harmony has prevailed and still prevails among its members.

Your Committee is heartily in accord with the President's recommendation, looking to the education of the masses in the elements of pure Homœopathy, and recommend that members be invited to submit tracts and leaflets to the Committee on Publication, which shall report such as they approve to the Association, and such as it shall approve shall bear its imprint and be sold to members in such quantities as they shall desire at actual cost.

In conclusion, we submit the following: that the Association tender a vote of thanks to Dr. Bell for his able and masterly statements as to our present condition, and for the encouragement which he demonstrates for persistent and progressive effort.

J. B. Gregg Custis,
Allen B. Carr,
B. L. B. Baylies.

The report was accepted and the recommendations adopted.

